# Investigating donor human milk composition globally to develop effective strategies for the nutritional care of preterm infants: Study protocol

**DOI:** 10.1371/journal.pone.0283846

**Published:** 2023-04-05

**Authors:** Maryanne T. Perrin, Kimberly Mansen, Kiersten Israel-Ballard, Scott Richter, Lars Bode, Daniela Hampel, Setareh Shahab-Ferdows, Lindsay H. Allen, Francisca Cofré Maggio, Emily Njuguna, Hoang Thi Tran, Aleksandra Wesolowska

**Affiliations:** 1 Department of Nutrition, University of North Carolina Greensboro, Greensboro, NC, United States of America; 2 Maternal, Newborn, Child Health and Nutrition, PATH, Seattle, WA, United States of America; 3 Department of Mathematics and Statistics, University of North Carolina Greensboro, Greensboro, NC, United States of America; 4 Department of Pediatrics and Mother-Milk-Infant Center of Research Excellence (MOMI CORE), University of California San Diego, San Diego, CA, United States of America; 5 United States Department of Agriculture/Agriculture Research Service, Western Human Nutrition Research Center, Davis, CA, United States of America; 6 Department of Nutrition, University of California Davis, Davis, CA, United States of America; 7 Neonatology Service, Hospital Sotero Del Rio, Santiago, Chile; 8 Pumwani Maternity and Referral Hospital, Nairobi, Kenya; 9 Human Milk Bank at Da Nang Hospital for Women and Children, Da Nang, Vietnam; 10 Department of Pediatrics, School of Medicine and Pharmacy, Da Nang University, Da Nang, Vietnam; 11 Department of Medical Biology, Laboratory of Human Milk and Lactation Research at Regional Human Milk Bank in Holy Family Hospital, Medical University of Warsaw, Warsaw, Poland; University of Agriculture Faisalabad, PAKISTAN

## Abstract

**Background:**

Globally, almost 15 million infants are born prematurely each year, disproportionately affecting low and middle-income countries. In the absence of mother’s milk, the World Health Organization recommends using donor human milk (DHM) due to its protective effect against necrotizing enterocolitis, a life-threatening intestinal disorder. The use of DHM is increasing globally, with many low and middle-income countries integrating donor milk banks into their public health strategies to reduce neonatal mortality, yet very little is known about the nutritional composition of DHM. Additional knowledge gaps include how DHM composition is influenced by milk banking practices, and whether preterm nutrient recommendations are achieved when DHM is used with commercially available fortifiers.

**Methods:**

We designed a multi-site study with eight geographically diverse milk bank partners in high, middle, and low-income settings that will examine and compare a broad range of nutrients and bioactive factors in human milk from 600 approved milk bank donors around the world to create comprehensive, geographically diverse nutrient profiles for DHM. We will then simulate the random pooling of 2 to 10 donors to evaluate the impact of pooling as a potential strategy for milk banks to manage nutrient variability in DHM. Finally, we will evaluate whether commercially available fortifiers meet nutrient recommendations when used with DHM.

**Discussion:**

We expect that results from this study will improve nutritional care globally for the growing number of preterm infants who receive donor human milk.

## Introduction

Donor human milk (DHM) from a human milk bank is recommended for preterm infants when mother’s own milk (MOM) is unavailable or insufficient [1–3]. This is based on evidence of reduced rates of necrotizing enterocolitis (NEC), a life-threatening intestinal disorder, when feeding DHM compared to preterm infant formula [4]. Globally, an estimated 800,000 infants receive DHM annually, supported by a growing network of over 750 human milk banks [5]. However, the composition of DHM has never been systematically studied [6], leaving critical knowledge gaps that impede the nutritional care of preterm infants. Further, emerging evidence suggests that how milk banks pool donors (combining the milk from multiple donors versus combining the milk from a single donor) influences the nutritional composition of the final DHM product used to feed infants, and that fortified DHM frequently does not reach preterm recommendations for some nutrients, further highlighting the need for systematic research into DHM production and clinical use [7, 8].

Evidence regarding differences in preterm infant growth outcomes based on primary feeding type (MOM versus DHM) is limited to observational studies due to ethical issues of withholding MOM. Results of these observational studies are conflicting, with some studies reporting inferior growth with DHM feeding [9–13], and other studies showing no difference [14–16]. Inconclusive findings may be related to differences in fortification protocols and the underlying nutritional composition of human milk, which is not frequently measured or reported. In a study that used an individualized fortification protocol based on nutritional analysis of human milk, preterm infants receiving fortified MOM (n = 37) had significantly greater weight, length, and head circumference Z-score gains than those receiving fortified DHM (n = 33), despite no significant difference in the macronutrients and energy delivered, suggesting suboptimal concentrations of nutrients other than macronutrients may be contributing to poor growth [17].

While human milk banks worldwide share some similarities in collecting, processing, storing, testing, and distributing DHM, they use a range of practices and implementation models due to a lack of global guidelines and standards [18]. These distinct practices may lead to geographic differences in the nutritional composition of DHM provided to infants. To address knowledge gaps related to donor human milk composition we have developed a multi-site study with three overarching objectives: (1) assess and compare the macronutrients, energy, vitamins, minerals, lactoferrin, IgA, and oligosaccharides in donated milk from approved donors to human milk banks from distinct global regions; (2) characterize how the number of donors combined into a pool during processing influences composition of nutrients in DHM by simulating the random pooling of donors; and (3) evaluate the effectiveness of commercially available human milk fortifiers at achieving macronutrient, vitamin, and mineral recommendations when used with DHM. We hypothesize that there will be differences in the variability of nutrients (e.g., 3-fold or greater differences in fat and some vitamins and minerals; limited differences in lactose), as well as geographic differences related to milk banking practices (e.g., the proportion of donations from donors having given birth prematurely versus at term). Further, we hypothesize that to reduce fat and micronutrient variability, more donors will be required per pool than to reduce protein and lactose variability. Finally, we hypothesize that macronutrients and micronutrients will be identified for enhancement in DHM-specific fortifiers and will differ by milk bank geography.

## Methods

### Study design and setting

This observational study will be conducted in established donor human milk banks operating in low-, middle- and high-income settings. We use a sampling lens of “approved donors,” which will allow us to collect milk reflective of what is available in a milk bank setting; therefore, no additional criteria related to donors will be applied. The donor approval process varies geographically, but typically includes an oral or written interview, screening for general health and medication use, and serological testing [18]. Milk bank sites were selected pragmatically based on geographic and economic diversity and the ability to collect the required number of samples within the study timeline. We are establishing a biorepository of DHM samples collected from approved donors to four accredited milk banks within the Human Milk Banking Association of North America (HMBANA) network: Mid-Atlantic Mothers’ Milk Bank provided through the Human Milk Science Institute and Biobank (Pittsburgh, Pennsylvania, USA); Mothers’ Milk Bank of North Texas (Benbrook, Texas, USA); Mothers’ Milk Bank of the Western Great Lakes (Elk Grove Village, Illinois, USA); and Northwest Mothers Milk Bank (Tigard, Oregon, USA). In addition, samples will be collected from approved milk bank donors at: Pumwani Maternity and Referral Hospital Human Milk Bank (Nairobi, Kenya); Da Nang Hospital for Women and Children Learning & Research Center for Human Milk and Newborn Care (Da Nang, Viet Nam); Banco de Leche Hospital Sótero del Río (Santiago, Chile); and Milk Bank at Mother’s Memorial Hospital Research, Lodz Institute (Lodz, Poland).

### Informed consent and ethical review

This study was reviewed by the Institutional Review Board at the University of North Carolina Greensboro and classified as non-human subjects research (protocol IRB-FY21-68). We will be collecting de-identified biospecimens from milk banks and will not have any interaction with the donors. While this study has been classified as non-human subjects research, milk bank donors do provide written consent that their donated milk may be used for research purposes as part of the normal donor screening and approval process that is conducted at each milk bank.

### Sample size

The number of samples for the biorepository was determined based on the underlying objectives. For our first objective, to characterize and compare DHM across diverse geographic areas, our minimum sample size was determined based on evidence from the INSPIRE study that country-level differences in HMOs were detected with 40–43 samples per region so we will collect a minimum of 50 samples per country [19]. For our second objective, to simulate the impact of donor pooling, we will only use samples from US donors due to the limited pooling of multiple donors in other countries [18]. We will collect samples reflective of what would be available for DHM production during a 1 to 2-month window which represents 8–16% of annual donations. Our US milk bank partners indicate that a 1 to 2-month production window translates to approximately 100 new donors; therefore, we will collect samples from 400 approved donors in the US (100 per milk bank). This sample size will provide a large enough database for simulating the impact of different random pooling scenarios (described in more detail in the section on statistical analysis). It also exceeds the sample size estimated to allow reporting of 95% confidence intervals at the 5^th^ and 10^th^ centiles without overlap (n = 255) [20], allowing us to provide robust estimates on the composition of human milk from US donors.

### Sample collection and handling

Each sample in the biorepository will represent milk from a unique, approved donor; therefore, study samples will reflect the way that donors are screened, and milk is handled within each milk bank setting. Briefly, milk banks typically sort an individual donor’s milk donations by the date that the milk was expressed and group these together into a Single Donor Pool. Single Donor Pools can range from a few ounces to hundreds of ounces, depending on whether milk banks have a minimum donation volume, and the frequency of donation and donor milk processing. Thus, a Single Donor Pool represents raw milk expressed over days or weeks from a single approved donor. To ensure that milk banks operating on a small scale provide samples that reflect a range of an individual donor’s milk, additional requirements include that the Single Donor Pool contains milk from at least three different pumping sessions collected over at least three calendar days and does not include colostrum (defined as the first five days postpartum). Milk banks will be provided with 20mL secure specimen containers and transfer pipettes. Sample collection will involve drawing 20mL from a well-mixed, Single Donor Pool and completing an entry on the collection log. Data collected for each sample will include sample ID, donor ID, first expression date in the pool, last expression date in the pool, infant month and year of birth, maternal year of birth, birth type (preterm or term), maternal COVID vaccine status (yes, no, unknown), and donor lifetime donation volume. Once a sample is collected from a donor, milk from that donor will not be collected again so that all samples in the biorepository are from unique donors.

**Fig 1:**
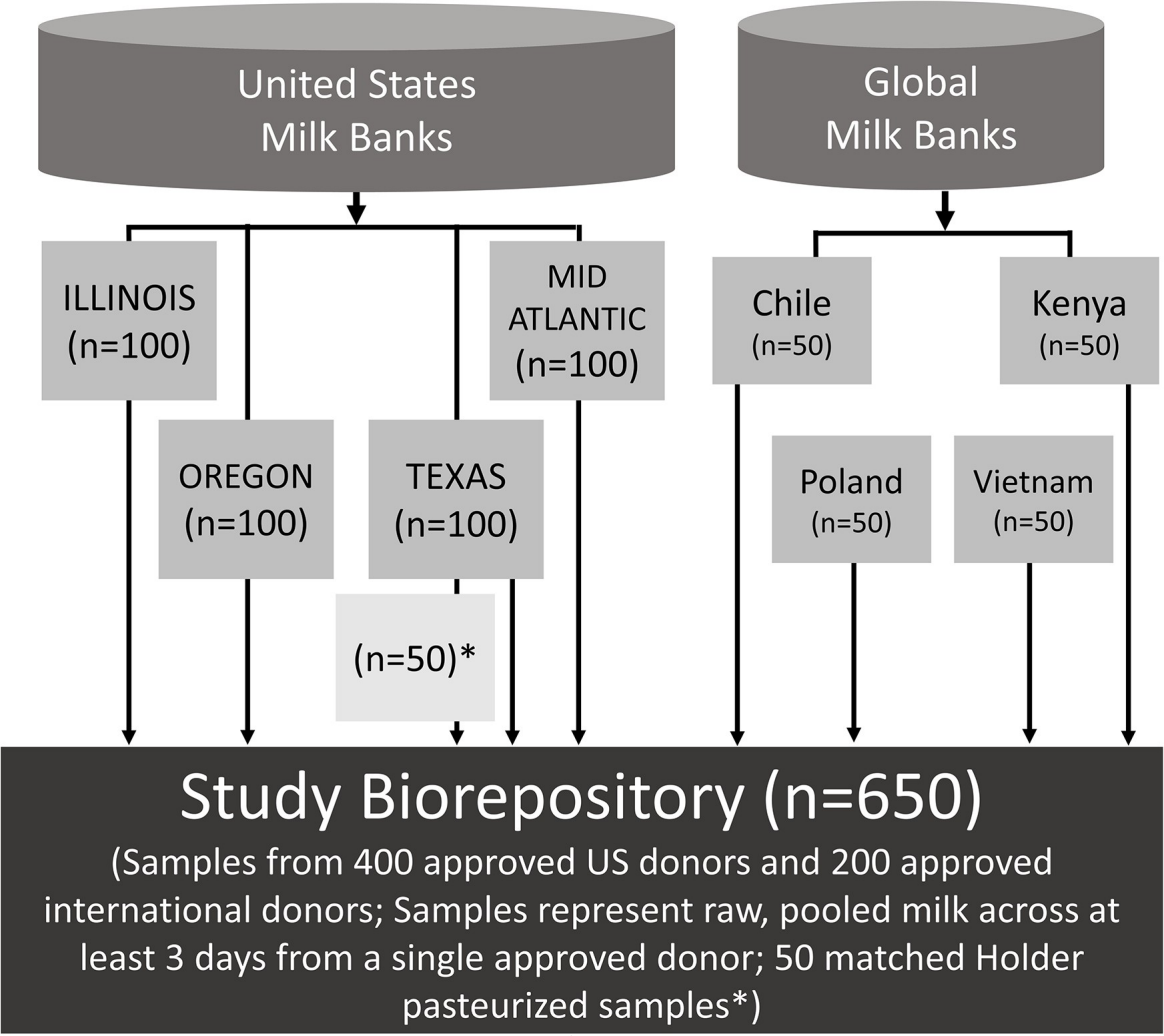
Overview of the global donor human milk biorepository.

The goal of this study is to report the expected nutrient concentrations of DHM after Holder pasteurization; however, in order to use the same sample collection protocol across all study site, we will be collecting raw milk samples from Single Donor Pools. To account for the expected nutrient loss that occurs during Holder pasteurization, we will also collect 50 post-pasteurization samples matched to a subset (n = 50) of the Single Donor Pools collected at the Mothers’ Milk Bank of North Texas. This approach is justified because all milk banking partners in this study use Holder pasteurization, the predominant processing method used globally in milk banks [18]. The literature on the impact of Holder pasteurization on donor milk nutrients is often limited to single studies [21], using micro-volumes of milk and a small number of samples. Our plan to account for the impact of pasteurization done in volumes reflective of actual milk banking practices adds rigor to our research methods. It will allow us to adjust nutrient values in our modeling to reflect expected loss during pasteurization. **Fig 1** provides an overview of the establishment of the DHM biorepository.

All study samples will be shipped on dry ice to the University of North Carolina Greensboro for initial processing. Samples collected at international milk banks will be monitored during shipment and replenished with dry ice to ensure maintenance of the cold chain during transport. To ensure representative aliquots for analysis, we will warm samples to 30-35°C in a shaking dry bath, stir samples on a magnetic mixer for five minutes, aliquot into 0.5 to 10mL volumes, and store in a -80°C freezer until analysis.

### Donor human milk analysis

All samples in the biorepository (n = 650) will be assessed for the macronutrients, vitamins, minerals, and bioactive factors summarized in **Table 1**. Unused samples will be maintained at the University of North Carolina Greensboro in a -80°C freezer for potential future analyses. Assays will be performed in replicates or a subset of blinded replicates by laboratories with expertise in the methods. Purified antibodies and enzyme-linked immunosorbent assay (ELISA) kits will be purchased from the same manufacturer and the same lot number to ensure consistency. A quality control human milk sample will be run on all ELISA plates as a method to monitor and report between-plate variability.

**Table 1:** Summary of analytes and methods of analysis for donor human milk samples.

Analyte	Method of Analysis	Location of Analysis
Total Fat	Mojonnier ether extraction	UNCG
Crude and True Protein	Kjeldahl (AOAC 991.20, 991.21, and 991.23)	Eurofins
Lactose	Megazyme enzymatic method (AOAC 2006.06)	UNCG
Energy	Atwater’s metabolizable energy conversions using 9 kcal/g fat, 4 kcal/g true protein, 4 kcal/g lactose, and 2 kcal/g HMOs	UNCG
Human milk oligosaccharides	High pressure liquid chromatography with fluorescence detection	UC-SD
Minerals (calcium, copper, iron, magnesium, manganese, phosphorus, potassium, selenium, sodium, and zinc)	Inductive coupled plasma mass spectrometry (ICP-MS)	USDA Western Human Nutrition Research Center
Thiamin, thiamin monophosphate, and thiamin diphosphate	Liquid chromatography coupled with fluorescence detection (HPLC-FLD)	USDA Western Human Nutrition Research Center
Riboflavin, flavin mononucleotide (FMN), flavin adenine dinucleotide (FAD), nicotinamide, nicotinamide mononucleotide (NMN), nicotinamide adenine dinucleotide (NAD), nicotinamide ribose, pyridoxal, pyridoxine, pyridoxamine, pyridoxal phosphate, biotin, pantothenic acid, choline, phosphocholine, and glycerophosphocholine	Liquid chromatography tandem mass spectrometry (UHPLC-MS/MS).	USDA Western Human Nutrition Research Center
Vitamin A and E vitamers (retinol, α-carotene, β-carotene, lutein/zeaxanthin, lycopene, β-cryptoxanthin, α-tocopherol, and γ-tocopherol)	Liquid chromatography with multiple wavelength detection (HPLC-MWL)	USDA Western Human Nutrition Research Center
Vitamin B12	IMMULITE®/IMMULTE® 1000 B12 competitive protein binding assays	USDA Western Human Nutrition Research Center
Immunoglobulin A	Enzyme-linked immunosorbent assay	UNCG
Lactoferrin	Enzyme-linked immunosorbent assay	UNCG

Notes: AOAC–Association of Analytical Chemists; HMO–human milk oligosaccharides; UC-SD–University of California, San Diego; UNCG–University of North Carolina Greensboro; USDA–United State Department of Agriculture

### Data collection and management

De-identified study data from the eight milk banks and multiple analytical labs will be aggregated and stored on a secure server. At the completion of the study, data will be made available via the National Institute of Child Health and Human Development (NICHD) Data Specimen Hub (DASH).

### Statistical analysis

Descriptive statistics will be computed for all milk analytes. Regression analysis will be used to estimate relationships between gestation term, lactation stage, donation volume, and nutrient composition. Evidence of nutrient differences in pre- and post- pasteurization samples will be assessed using a paired t-test. Differences in nutrient composition between geographic regions will be evaluated with ANOVA with a Tukey’s test for multiple comparisons, after adjusting all values to reflect the impact of pasteurization.

Our donor milk nutrient database from samples collected from 400 unique US donors will serve as the population for selecting donors for a simulation to model the impact of the number of donors used per DHM pool. One thousand (1000) pools will be generated using Monte Carlo sampling based on the random combination of donors. Nine separate simulations will be conducted, each representing a different number of donors (2 to 10) randomly selected for a pool. The maximum value of 10 donors per pool reflects potential production constraints and balancing the risk for potential recall (the more donors per pool, the more pools would be impacted should DHM need to be recalled based on issues with an individual donor). The simulations will be weighted to account for lifetime donation volumes so that a donor with higher lifetime donation volume will be selected more frequently in the simulation than a donor with a lower lifetime donation volume. Pooling scenarios (e.g., 2 to 10 donors) will be considered appropriate if 80% of the pools meet key nutrient thresholds described below. The 80% threshold was selected because approximately 20% of DHM in the US is used in home environments with less medically vulnerable populations [22]. The nutrient scenarios that will be evaluated include: (1) 0.9 g/dL of true protein; (2) 3.5 g/dL of fat; and (3) 210 μg/mL of the human milk oligosaccharide (HMO) disialyllacto-N-tetraose (DSLNT). The rationale for selecting these nutrients is that improved weight, length, and head circumference growth has been reported in preterm infants with greater protein intake [23]; fat is the most variable macronutrient in DHM and a major contributor to energy, with 3.5 g/dL of fat leading to 19–20 kcal/ounce DHM [24, 25]; and DSLNT in human milk at the threshold dose is associated with reduced rates of necrotizing enterocolitis [26, 27].

To evaluate the impact of fortifiers on DHM, we will work with medical directors at our US and international partner milk banks to identify multi-nutrient fortifiers commonly used with DHM so that analyses reflect available fortifier products and practices. The expected nutrient composition for fortified DHM will be computed based on the manufacturer’s nutrient profile of the multi-nutrient fortifiers identified. Computations will reflect the mixing instructions provided by the manufacturer (e.g., 25 mL of DHM to 5 mL of fortifier) or as described by the local clinician, if different. Theoretical pooled samples from the optimum distribution identified during our pooling simulation will be used for US DHM values since it is common practice to pool multiple donors in US milk banks [28]. For international milk banks, individual donor samples will be used due to limited practices of pooling multiple donors outside of the US. We will compare the nutrients delivered using DHM with setting-specific fortification practices to recommendations established by ESPGHAN for preterm infants [29]. We will assume full feeding volumes of 160 mL/kg/day and use the mid-point when ESPGHAN recommendations are provided as ranges. Full-feed nutrient values achieved using fortified DHM will be reported as a percentage of recommendation (calculated as nutrients delivered at full-feeds using fortified DHM divided by ESPGHAN nutrient recommendations); percentages less than 100% will indicate nutrient goals that were not achieved with fortification.

## Discussion

Here we describe the methodology for a multi-site, international study that will: (1) characterize the composition of over 50 nutrients and bioactive compounds in breast milk provided by 600 approved donors to eight geographically and economically diverse milk banks; (2) simulate the impact of randomly pooling up to 10 donors to identify potential strategies for milk banks to control the variability of key nutrients; and (3) evaluate the impact of existing, geographically relevant fortification strategies to determine if they meet nutrient recommendations when used with DHM.

Our study has several strengths. It will be the first multi-country study on DHM composition and reflects a variety of milk bank models (e.g., different gestation stages of donors; different lactation stages of donations). DHM samples will be collected systematically from milk banks in diverse geographic and economic settings and will reflect the methods that milk banks use when processing a donor’s milk. We will use what are considered the most robust analytical methods for assessing human milk [30], which have not often been used when reporting the nutrient composition of DHM [6]. Vitamin and mineral analysis will occur at the USDA lab that is conducting a multi-site, international study to develop reference values for mother’s milk [20]; therefore, we will be able to directly compare our DHM values to the newly emerging reference values for mother’s milk. Several micronutrients that have not previously been reported for DHM will be assessed, including selenium, thiamin, riboflavin, niacin, and B12. We will use computer modeling techniques to explore how milk banks can reduce nutrient variability, and the adequacy of existing fortifiers when used with DHM, with the ultimate goal of improving nutritional therapies for the preterm infant. Finally, we will collect donor COVID-19 vaccination status as a potential exploratory variable for future analysis.
